# Isolated Robotic Mitral Valve Replacement Across Body Mass Index Categories: Early Perioperative Outcomes

**DOI:** 10.3390/jcm15197756

**Published:** 2026-10-08

**Authors:** Zinar Apaydın, Barış Timur, Batuhan Yazıcı, Mustafa Can Kaplan, Salih Güler, İsa Can, Tural Muradlı, Safa Göde, Ersin Kadiroğulları

**Affiliations:** 1 Department of Cardiovascular Surgery, Mehmet Akif Ersoy Thoracic and Cardiovascular Surgery Training and Research Hospital, İstanbul 34303, Türkiye; dr.baristimur@gmail.com (B.T.); dr.batuhanyazici@gmail.com (B.Y.); drsalihguler@gmail.com (S.G.); iscan3181@gmail.com (İ.C.); turalmuradli@gmail.com (T.M.); safagode@yahoo.com (S.G.); ersinkadirogullari@gmail.com (E.K.); 2 Department of Cardiovascular Surgery, TekirdagNamık Kemal University, Tekirdag 59030, Türkiye; mecankaplan@gmail.com

**Keywords:** obesity, obesity paradox, robotic cardiac surgery, mitral valve surgery, mitral valve replacement

## Abstract

**Background**: Obesity may pose additional technical and perioperative challenges in robotic mitral valve surgery. This study evaluated early perioperative outcomes of isolated robotic mitral valve replacement (MVR) across BMI categories. **Methods**: We retrospectively analyzed 146 patients who underwent isolated robotic MVR at a single center, categorized into BMI categories of <25 kg/m^2^ (n = 60), 25.0–29.9 kg/m^2^ (n = 38), or ≥30 kg/m^2^ (n = 48). Preoperative characteristics, operative variables, and early postoperative outcomes were compared among groups. The study was exploratory. Four clinically selected outcomes, cardiopulmonary bypass time, ventilation duration, hospital length of stay, and postoperative left ventricular ejection fraction, were evaluated using adjusted median quantile regression with bootstrap 95% confidence intervals, and multiplicity across postoperative outcomes was addressed using the Benjamini–Hochberg false discovery rate. **Results**: Patients with BMI ≥ 30 kg/m^2^ were older and had a higher prevalence of diabetes and higher EuroSCORE II values. Unadjusted comparisons of cardiopulmonary bypass and aortic cross-clamp times, conversion to sternotomy, ventilation duration, intensive care unit and hospital length of stay, drainage volume, and transfusion requirements yielded *p* values > 0.05. In adjusted analyses of the four selected outcomes, all 95% confidence intervals for the BMI-group estimates included zero. Pneumonia was numerically more frequent in patients with BMI ≥ 30 kg/m^2^ (10.4% vs. 2.6% and 1.7%), but the estimates were imprecise. In-hospital mortality was low, with one death overall. Only eight patients had BMI ≥ 35 kg/m^2^. **Conclusions**: Among patients considered suitable for robotic MVR, the adjusted estimates showed no statistically detectable association between BMI category and the selected early outcomes. However, the confidence intervals did not establish equivalence, and the findings apply only to selected robotic surgical candidates.

## 1. Introduction

Obesity remains a major global health challenge and is associated with increased cardiovascular morbidity and mortality. Body mass index (BMI) is widely used to classify patients according to weight status, with obesity conventionally defined as a BMI ≥ 30 kg/m^2^ [1,2,3].

Historically, obesity has been linked to worse outcomes after cardiac surgery [4]. However, multiple studies have reported that patients who are overweight or have Class I obesity may experience outcomes comparable to, or occasionally better than, those of normal-weight patients, a phenomenon termed the “obesity paradox” [5,6,7,8,9].

With advances in minimally invasive and robotic techniques, less invasive mitral valve operations have become increasingly common [10]. Nonetheless, robotic surgery in patients with higher BMI can present technical challenges, including access limitations and reduced robotic arm maneuverability due to increased subcutaneous tissue. In high-risk or anatomically challenging patients, treatment selection may also include transcatheter mitral interventions; however, transcatheter mitral valve implantation remains limited by anatomical suitability, device-related constraints, and risks such as left ventricular outflow tract obstruction [11].

In conventional cardiac surgery, obesity may increase the difficulty of operative exposure and is associated with impaired respiratory mechanics, wound complications, and more challenging vascular access [4]. In robotic cardiac surgery, the smaller access incisions may reduce wound-related morbidity, but obesity can still affect port placement, robotic instrument maneuverability, peripheral cannulation, and the need for conversion to sternotomy [12,13]. Because evidence regarding the influence of BMI on robotic mitral valve replacement remains limited, particularly in replacement cohorts, these issues formed the clinical rationale for the present study. Against this background, we investigated the association between BMI category and perioperative outcomes in patients undergoing isolated robotic mitral valve replacement (MVR).

## 2. Materials and Methods

### 2.1. Study Design and Setting

This retrospective, single-center study was conducted at a tertiary referral cardiovascular surgery center and included patients undergoing isolated robotic mitral valve replacement (MVR) between March 2016 and April 2024. During the study period, 306 patients underwent robotic mitral valve surgery at our institution. Of these, 146 underwent isolated robotic MVR and constituted the present study cohort; the remaining 160 underwent mitral valve repair or concomitant major cardiac procedures and were therefore not eligible for the present analysis.

### 2.2. Ethics Statement

This retrospective study used anonymized clinical data collected during routine care and involved no study-specific intervention or additional data collection. Written informed consent for the use of clinical data for scientific research and academic publication was obtained from all patients before surgery. The study was conducted in accordance with the ethical principles of the Declaration of Helsinki.

### 2.3. Study Cohort and Patient Selection

To ensure a procedurally homogeneous study population, the present analysis was restricted to 146 patients undergoing isolated robotic MVR. In this study, isolated MVR was defined as MVR without concomitant major cardiac surgery; concomitant atrial fibrillation ablation was permitted. Mitral valve repair procedures were excluded because repair is a technically distinct operation with procedure-dependent and sometimes substantially shorter or longer cardiopulmonary bypass and aortic cross-clamp times; restricting the cohort to replacement therefore provided a more homogeneous procedural population for BMI comparison.

At our institution, candidacy for robotic mitral valve surgery is determined according to anatomical and clinical suitability. Patients with a history of previous cardiac or thoracic surgery, chest wall deformity or significant chest trauma, prior thoracic radiotherapy, a femoral artery diameter < 7 mm, or significant aortoiliac arterial disease are generally considered unsuitable for a robotic approach. These institutional criteria are applied when determining candidacy for robotic mitral valve surgery and are distinct from the study-specific cohort definition. BMI itself is not considered an absolute contraindication to the robotic approach. The present cohort therefore represents patients who were ultimately selected for the robotic approach rather than all patients referred for mitral valve surgery. A structured screening log of patients who were evaluated but not selected for robotic surgery was not available; consequently, the total number of excluded patients, their BMI distribution, and BMI-specific reasons for exclusion could not be reliably reconstructed.

### 2.4. Data Collection and Definitions

Demographic, preoperative, intraoperative, and postoperative data were retrospectively obtained from electronic medical records and patient charts. Preoperative variables included age, sex, BMI, body surface area, EuroSCORE II, New York Heart Association functional class, comorbidities, cardiac rhythm, and echocardiographic parameters. Intraoperative variables included cardiopulmonary bypass time, aortic cross-clamp time, prosthesis type, conversion to sternotomy, and concomitant atrial fibrillation ablation.

Postoperative variables included mechanical ventilation duration, intensive care unit length of stay, hospital length of stay, drainage volume, blood product utilization, postoperative atrial fibrillation, temporary and permanent pacemaker requirement, neurologic events, acute kidney injury, dialysis requirement, pericardial effusion or tamponade, pleural effusion, pneumonia, groin infection, pneumothorax, reoperation for bleeding, postoperative left ventricular ejection fraction, in-hospital mortality, and early readmission.

Mitral valve etiology was classified as rheumatic, mixed, Barlow, endocarditis, or etiology not otherwise specified based on the available clinical records. For descriptive reporting, mutually exclusive etiologic categories were assigned with priority to rheumatic disease, followed by mixed disease, Barlow disease, endocarditis, and etiology not otherwise specified. In three patients, the available clinical records did not permit reliable assignment to a specific etiologic category; these cases were therefore reported as etiology not otherwise specified.

In-hospital mortality was defined as death occurring during the index hospitalization. Postoperative pleural effusion and pneumothorax were recorded when they required intervention. Postoperative LVEF was defined as the value measured by transthoracic echocardiography using the biplane Simpson method before hospital discharge. Acute kidney injury was defined according to Kidney Disease: Improving Global Outcomes criteria as an increase in serum creatinine of ≥0.3 mg/dL within 48 h or ≥1.5 times the baseline value within 7 days. Pneumonia was defined as a new radiographic infiltrate accompanied by clinical evidence of infection and antibiotic treatment. A transient cerebrovascular event was defined as a focal neurological deficit resolving completely within 24 h, whereas a permanent cerebrovascular event was defined as a neurological deficit persisting for more than 24 h. Early readmission was defined as an unplanned hospital readmission within 30 days after discharge.

New-onset postoperative AF was defined as a documented episode of AF occurring during the index hospitalization in a patient without preoperative AF. During the intensive care unit stay, cardiac rhythm was continuously monitored by telemetry. After transfer to the ward, rhythm assessment was based on routine daily 12-lead electrocardiography and additional electrocardiograms obtained when clinically indicated. Because this was a retrospective study, postoperative rhythm outcomes were based on AF documented in the clinical record during the index hospitalization, and systematic post-discharge rhythm monitoring was not performed. Preoperative hypertension was defined as a documented history of hypertension and/or ongoing use of antihypertensive medication before surgery. Red blood cell transfusion was recorded as the total number of units administered during the perioperative period, including intraoperative transfusions.

### 2.5. BMI Classification

BMI was calculated as body weight in kilograms divided by the square of height in meters (kg/m^2^). For categorical analyses, patients were stratified into three BMI categories: <25 kg/m^2^ (n = 60), 25.0–29.9 kg/m^2^ (n = 38), and ≥30 kg/m^2^ (n = 48).

### 2.6. Surgical Procedure

**Figure 1:**
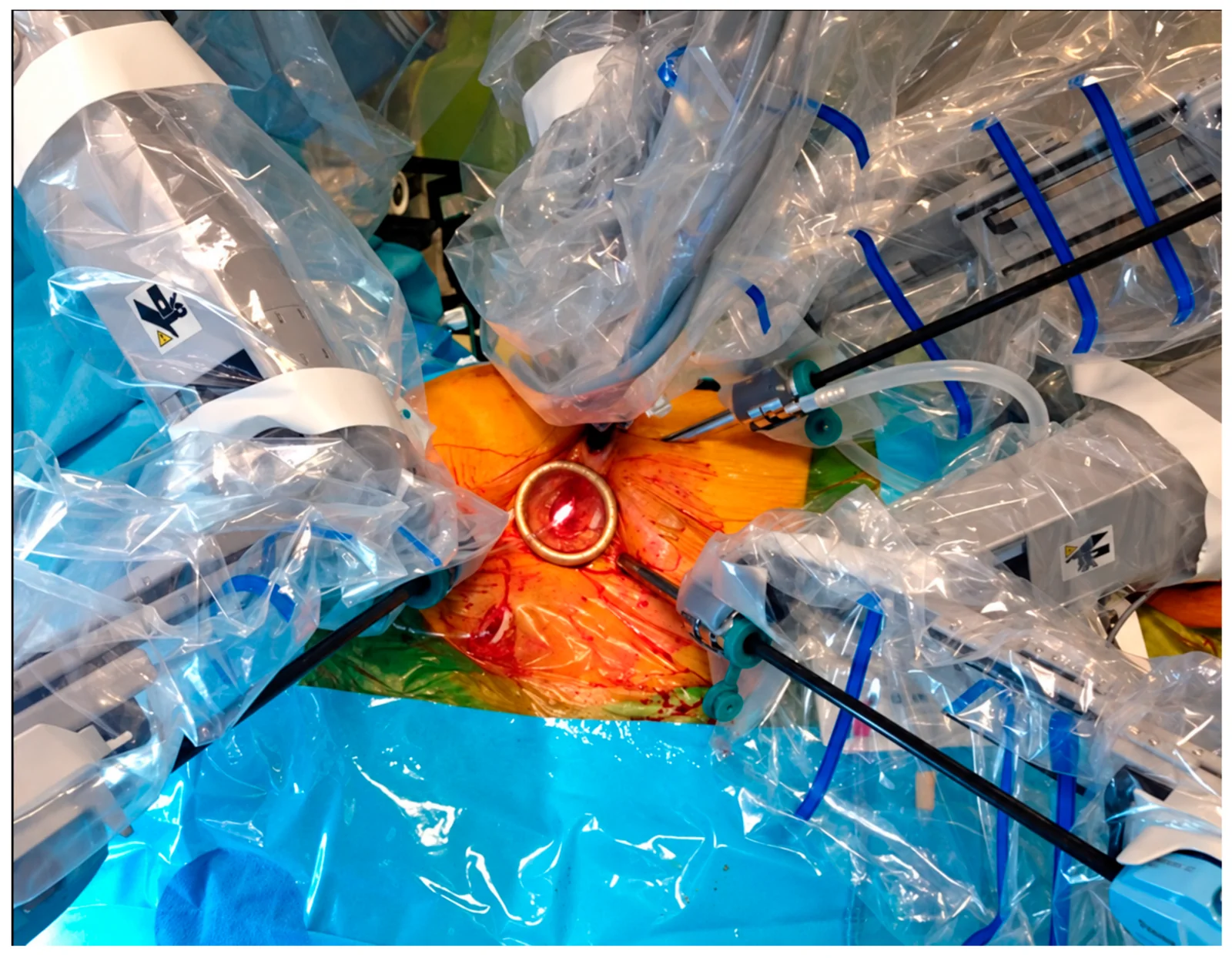
Intraoperative view of the robotic setup and port placement in a patient with a body mass index > 30 kg/m^2^, showing the docked da Vinci Si system and the right hemithorax working ports.

All procedures were performed under general anesthesia with a single-lumen endotracheal tube, and a transesophageal echocardiography (TEE) probe was placed after intubation. All operations were performed with the da Vinci Si surgical system (Intuitive Surgical Inc., Sunnyvale, CA, USA). Peripheral cardiopulmonary bypass (CPB) access was established by direct surgical cannulation of the right femoral artery and vein together with percutaneous cannulation of the right internal jugular vein. CPB was initiated after systemic heparinization. Ports were placed in the right hemithorax and the robotic system was docked (Figure 1). A limited pericardiotomy was performed under robotic visualization, preserving the integrity of the phrenic nerve, and the patient was cooled to 32 °C. The ascending aorta was exposed and vented through the aortic root. A transthoracic aortic cross-clamp (Chitwood clamp; Aesculap Inc., Center Valley, PA, USA) was applied through the second intercostal space, and diastolic arrest was achieved with antegrade cold Del Nido cardioplegia. After cardiac arrest, bicaval control was obtained and the interatrial (Sondergaard) groove was dissected to expose the left atrium. Left atriotomy was performed and a robotic atrial retractor was positioned to optimize exposure of the mitral valve apparatus. The subvalvular apparatus was preserved whenever technically feasible in order to maintain left ventricular geometry and support postoperative ventricular function. After excision of the diseased native valve, the prosthesis was implanted with everting pledgeted sutures in an anti-anatomical position under direct robotic visualization. The left atrium was then closed in the standard fashion. After de-airing, the cross-clamp was removed and the patient was rewarmed and gradually weaned from CPB, with cardiac and prosthetic valve function reassessed by TEE. Once hemodynamic parameters were satisfactory, CPB was discontinued and the robotic instruments were withdrawn. Hemostasis was achieved, chest drains were placed in the pericardial and right pleural spaces, and all port sites were closed in layers. The operative strategy was consistent throughout the study cohort, and all procedures were performed by the institutional robotic cardiac surgery team throughout the study period.

### 2.7. Statistical Analysis

Continuous variables were summarized as mean ± standard deviation (SD) when approximately normally distributed and as median with interquartile range (IQR) otherwise. Distributional characteristics were evaluated using the Shapiro–Wilk test together with assessment of skewness. Homogeneity of variance was assessed using Levene’s test.

For comparisons across the three BMI groups, normally distributed continuous variables with homogeneous variances were analyzed using one-way analysis of variance (ANOVA), whereas Welch’s ANOVA was used when the assumption of equal variances was violated. Non-normally distributed continuous variables were compared using the Kruskal–Wallis test; based on these distributional assessments, this approach was applied to all continuous intraoperative and postoperative variables. When an overall group difference was statistically significant, Games–Howell post hoc testing was used following Welch’s ANOVA and Dunn’s test with Holm adjustment was used following the Kruskal–Wallis test.

Categorical variables were presented as counts and percentages and compared across BMI groups using Fisher’s exact test. For categorical variables with a significant overall difference, pairwise Fisher’s exact tests with Holm correction for multiple comparisons were performed. All tests were two-sided, and a *p* value < 0.05 was considered statistically significant. Statistical analyses were performed using R version 4.5.3 (R Foundation for Statistical Computing, Vienna, Austria).

To evaluate potential temporal effects, the annual distribution of BMI categories was compared using a Monte Carlo Fisher–Freeman–Halton test with 1,000,000 resamples and a fixed random seed. The study period was additionally divided into an early institutional era (2016–2019) and a later era (2020–2024). BMI distribution across eras was assessed using the Fisher–Freeman–Halton exact test.

No single primary endpoint was prespecified. Given the retrospective design and the number of evaluated outcomes, all analyses were considered exploratory. To avoid selecting adjusted analyses solely according to univariable statistical significance, four clinically relevant outcomes were selected for adjusted modeling: cardiopulmonary bypass time, mechanical ventilation duration, hospital length of stay, and postoperative LVEF. Median (τ = 0.50) quantile regression was used because these outcomes were not normally distributed. Each model included BMI category, age, diabetes mellitus, preoperative LVEF, and procedural year. EuroSCORE II was not included in the adjusted models because its component structure overlaps with age, diabetes, and LVEF. The BMI < 25 kg/m^2^ group was the reference category. Adjusted median differences are reported with percentile-based 95% confidence intervals obtained from 2000 bootstrap replications. Two-sided bootstrap *p* values were calculated as 2 × min[P(β* ≤ 0), P(β* ≥ 0)] for each BMI coefficient. Missing-data patterns were assessed before analysis; no missing values were identified among variables included in Table 1, Table 2, Table 3 and Table 4 or the adjusted models, so no imputation was performed and analyses used complete cases. Multiplicity across the 20 postoperative endpoints in Table 3 was addressed using the Benjamini–Hochberg false discovery rate, with both unadjusted *p* values and adjusted q values reported. The Holm method remained applicable to pairwise post hoc comparisons following significant overall tests. For mortality, transient and permanent cerebrovascular events, dialysis, and conversion to sternotomy, event counts were considered insufficient for reliable multivariable modeling; these outcomes were therefore summarized using event proportions and exact Clopper–Pearson 95% confidence intervals. Absence of statistical significance was not interpreted as equivalence, and confidence intervals were used to describe the precision of the estimates. As a sensitivity analysis addressing the loss of information caused by categorization, BMI was additionally entered as a continuous variable in the same four adjusted quantile-regression models. Continuous-BMI estimates are reported per 5 kg/m^2^ increase, with 95% confidence intervals based on 2000 bootstrap replications.

## 3. Results

A total of 146 patients undergoing isolated robotic mitral valve replacement were included in the analysis. According to BMI, 60 patients (41.1%) had a BMI < 25 kg/m^2^, 38 (26.0%) had a BMI of 25.0–29.9 kg/m^2^, and 48 (32.9%) had a BMI ≥ 30 kg/m^2^ (Table 1). Among the 48 patients with BMI ≥ 30 kg/m^2^, 40 had Class I obesity (BMI 30.0–34.9 kg/m^2^), seven had Class II obesity (BMI 35.0–39.9 kg/m^2^), and one had Class III obesity (BMI ≥ 40 kg/m^2^).

The annual distribution of procedures according to BMI category is shown in Supplementary Table S1. BMI-group distribution did not differ significantly across calendar years (Monte Carlo Fisher–Freeman–Halton test with 1,000,000 resamples, *p* = 0.311) or between the early (2016–2019; n = 74) and later (2020–2024; n = 72) institutional eras (exact *p* = 0.137).

Preoperative characteristics according to BMI group are presented in Table 1. Patients with higher BMI were older, with mean ages of 43.3 ± 16.5, 48.0 ± 12.9, and 53.6 ± 11.5 years across the three groups (overall *p* = 0.001). Post hoc analysis demonstrated a significant difference between the BMI < 25 and BMI ≥ 30 kg/m^2^ groups (adjusted *p* < 0.001). As expected, body surface area increased progressively across BMI categories (1.70 ± 0.19, 1.84 ± 0.15, and 1.99 ± 0.14 m^2^; *p* < 0.001), with significant pairwise differences among all three groups (Table 1).

Rheumatic disease was the predominant etiology in all BMI groups, occurring in 44/60 (73.3%), 27/38 (71.1%), and 41/48 (85.4%) patients. Mixed disease occurred in 12 (20.0%), 10 (26.3%), and 5 (10.4%); Barlow disease in 2 (3.3%), 0, and 1 (2.1%); endocarditis in 0, 0, and 1 (2.1%); and etiology was not otherwise specified in 2 (3.3%), 1 (2.6%), and 0 patients (Table 1).

EuroSCORE II also differed across BMI groups (*p* = 0.019), with higher values in the BMI ≥ 30 kg/m^2^ group compared with the BMI < 25 kg/m^2^ group on post hoc analysis (adjusted *p* = 0.021). Diabetes mellitus was more prevalent in patients with BMI ≥ 30 kg/m^2^ (27.1%) than in those with BMI < 25 kg/m^2^ (8.3%) or BMI 25.0–29.9 kg/m^2^ (5.3%) (overall *p* = 0.007); pairwise differences remained significant for BMI ≥ 30 versus <25 kg/m^2^ (adjusted *p* = 0.035) and BMI ≥ 30 versus 25.0–29.9 kg/m^2^ (adjusted *p* = 0.029) (Table 1).

Preoperative LVEF differed across groups (overall *p* = 0.030), although the median was 60% in all three groups. Post hoc analysis identified a significant difference between the BMI < 25 and BMI ≥ 30 kg/m^2^ groups (adjusted *p* = 0.025). No significant differences were observed in sex, NYHA functional class, hypertension, coronary artery disease, chronic obstructive pulmonary disease, chronic kidney disease, dialysis, preoperative atrial fibrillation, LVEDD, left atrial diameter, or tricuspid regurgitation (Table 1).

Intraoperative characteristics are summarized in Table 2. Median CPB time was 154.5 (IQR, 133.2–184.2) minutes in the BMI < 25 kg/m^2^ group, 148 (119–181.5) minutes in the BMI 25.0–29.9 kg/m^2^ group, and 163 (127.5–202.2) minutes in the BMI ≥ 30 kg/m^2^ group (overall *p* = 0.401). Corresponding cross-clamp times were 102 (82.8–123.2), 92.5 (77.2–111.5), and 104 (85.5–127.8) minutes (overall *p* = 0.234). These *p* values should be interpreted together with the confidence intervals.

The overall comparison of prosthesis selection yielded *p* = 0.321. Conversion to sternotomy occurred in 1/60 patients with BMI < 25 kg/m^2^ (1.7%; exact 95% CI, 0.0–8.9), 1/38 with BMI 25.0–29.9 kg/m^2^ (2.6%; 0.1–13.8), and 3/48 with BMI ≥ 30 kg/m^2^ (6.2%; 1.3–17.2). Because only five conversions occurred, no adjusted model was fitted and the estimates should be interpreted descriptively. Concomitant atrial fibrillation ablation was performed in 10.0%, 7.9%, and 6.2% of patients (overall *p* = 0.926). The recorded reasons for conversion included extensive pleural adhesions and bleeding that could not be controlled through the robotic approach.

Postoperative outcomes are presented in Table 3. The unadjusted comparisons did not identify statistically significant differences in mechanical ventilation duration (*p* = 0.181), ICU length of stay (*p* = 0.635), hospital length of stay (*p* = 0.792), postoperative drainage (*p* = 0.690), or red blood cell transfusion requirements (*p* = 0.934). After false-discovery-rate adjustment across the 20 postoperative endpoints, none met the q < 0.05 threshold. These findings indicate an absence of statistically detectable differences in this sample; the confidence intervals should be considered when judging precision.

Postoperative pneumonia occurred in 1/60 (1.7%), 1/38 (2.6%), and 5/48 (10.4%) patients across increasing BMI categories. The absolute difference between the BMI ≥ 30 kg/m^2^ and BMI < 25 kg/m^2^ groups was 8.7 percentage points (95% CI, −1.0 to 18.6). Among patients with pneumonia, median mechanical ventilation duration was 10 h (IQR, 9–55), compared with 10 h (IQR, 8–12) among patients without pneumonia.

The proportion of patients receiving at least one red blood cell unit was 35/60 (58.3%) in the BMI < 25 kg/m^2^ group, 20/38 (52.6%) in the BMI 25.0–29.9 kg/m^2^ group, and 27/48 (56.3%) in the BMI ≥ 30 kg/m^2^ group (overall, 82/146 [56.2%]).

The unadjusted postoperative LVEF comparison yielded *p* = 0.038, with median values of 60% (IQR, 55–60), 60% (55–60), and 55% (53.75–60) across increasing BMI categories. This result did not remain statistically significant after false-discovery-rate adjustment (q = 0.765). Adjusted median differences for the four clinically selected outcomes are presented in Table 4. Relative to BMI < 25 kg/m^2^, the adjusted median differences were 0.20 min (95% CI, −32.56 to 20.36) for BMI 25.0–29.9 kg/m^2^ and 7.32 min (−25.93 to 37.31) for BMI ≥ 30 kg/m^2^ for cardiopulmonary bypass time; −0.58 h (−1.85 to 0.00) and −0.81 h (−3.00 to 0.48) for ventilation duration; 0.00 days (−1.33 to 1.00) and 0.00 days (−0.76 to 1.26) for hospital length of stay; and 0.20 percentage points (−3.60 to 2.79) and −2.00 percentage points (−5.00 to 1.13) for postoperative LVEF. All confidence intervals included zero. In the continuous-BMI sensitivity analysis, each 5 kg/m^2^ increase in BMI corresponded to adjusted median changes of 2.91 min in cardiopulmonary bypass time (95% CI, −10.91 to 14.22), −0.50 h in ventilation duration (−1.29 to 0.10), 0.00 days in hospital length of stay (−0.37 to 0.49), and −0.78 percentage points in postoperative LVEF (−1.73 to 0.63). All confidence intervals included zero (Table 4, Panel C).

Event counts for cerebrovascular events, dialysis, and other major complications were low. Accordingly, no adjusted regression models were fitted for these outcomes. Exact descriptive estimates are presented in Table 4. In the overall cohort, transient cerebrovascular events occurred in 2/146 patients (1.4%; 95% CI, 0.2–4.9), permanent cerebrovascular events in 1/146 (0.7%; 95% CI, 0.0–3.8), dialysis in 2/146 (1.4%; 95% CI, 0.2–4.9), and conversion to sternotomy in 5/146 (3.4%; 95% CI, 1.1–7.8). No groin infection was recorded in the cohort (0/146); other femoral access complications were not separately coded in the retrospective database.

In-hospital mortality occurred in 1/146 patients (0.7%; exact 95% CI, 0.0–3.8). The death occurred in the BMI < 25 kg/m^2^ group; the group-specific confidence intervals were wide because of the very small number of events. These descriptive data do not permit inference regarding a BMI-related mortality effect.

## 4. Discussion

This retrospective, single-center exploratory study aimed to evaluate the association between BMI category and early perioperative outcomes in 146 patients undergoing isolated robotic mitral valve replacement. Despite older age, a higher prevalence of diabetes, and higher EuroSCORE II values among patients with BMI ≥ 30 kg/m^2^, adjusted analyses did not identify statistically detectable associations between BMI category and cardiopulmonary bypass time, ventilation duration, hospital length of stay, or postoperative LVEF. Pneumonia was numerically more frequent in the highest BMI category, while in-hospital mortality was low, with one death overall.

The 95% confidence intervals for all adjusted BMI estimates included zero, but several were sufficiently wide to remain compatible with clinically relevant differences. The results therefore should not be interpreted as demonstrating equivalence or the absence of a BMI effect. Patients with BMI ≥ 30 kg/m^2^ were older and had a higher prevalence of diabetes and higher EuroSCORE II values, emphasizing the importance of covariate adjustment. EuroSCORE II is a widely used operative risk model and has also been validated in patients undergoing minimally invasive mitral valve surgery [14,15]. In-hospital mortality was rare and was evaluated descriptively rather than through multivariable modeling.

The etiology distribution indicates that this cohort was predominantly rheumatic rather than representative of contemporary degenerative mitral regurgitation cohorts. Rheumatic disease was present in 112/146 (76.7%) patients; mixed disease, Barlow disease, endocarditis, and unspecified etiology accounted for the remaining cases. This distinction should be considered when comparing these findings with series focused on degenerative mitral regurgitation repair.

Obesity has traditionally been regarded as an important risk factor in cardiac surgery, owing to its associations with metabolic disease, impaired respiratory mechanics, wound complications, and technical difficulty during operative exposure [4]. These considerations may be particularly relevant when evaluating obese patients for minimally invasive and robotic cardiac procedures [12,13]. The relationship between BMI and outcomes in cardiovascular disease and cardiac surgery remains a matter of ongoing debate [6,7,8]. Prior studies have reported divergent findings. Some suggest comparable or more favorable outcomes among overweight and obese patients, whereas others report increased morbidity depending on the procedure, the severity of obesity, and the endpoint definitions applied [9,12,13]. Within robotic cardiac surgery specifically, prior reports indicate that obesity does not necessarily increase perioperative morbidity or mortality and that robotic mitral valve procedures can be performed safely in selected obese patients. A recent study examining long-term outcomes after surgical repair of degenerative mitral regurgitation similarly found no adverse association between elevated BMI and survival [16], suggesting that the relationship between BMI and outcomes may also differ within mitral valve surgical populations. Operative duration is a clinically relevant measure of technical burden in robotic mitral surgery. In the adjusted analysis, the median CPB-time differences relative to BMI < 25 kg/m^2^ were 0.20 min (95% CI, −32.56 to 20.36) for BMI 25.0–29.9 kg/m^2^ and 7.32 min (−25.93 to 37.31) for BMI ≥ 30 kg/m^2^. These confidence intervals included zero but were wide and do not exclude potentially meaningful shorter or longer operative times. Conversion to sternotomy was numerically more frequent in the BMI ≥ 30 kg/m^2^ group, but only five events occurred and the group-specific confidence intervals were imprecise. Accordingly, these estimates should be interpreted cautiously given the limited precision.

Unadjusted comparisons did not identify statistically significant BMI-group differences in ventilation duration, ICU or hospital length of stay, drainage, or red blood cell transfusion. In the adjusted models, the confidence intervals for ventilation duration and hospital length of stay included zero. These estimates were imprecise, particularly given the modest sample size and the width of the confidence intervals.

Postoperative LVEF yielded the smallest unadjusted *p* value among the postoperative outcomes (*p* = 0.038), but this finding did not remain significant after false-discovery-rate adjustment (q = 0.765). In the clinically selected adjusted model, the BMI ≥ 30 kg/m^2^ estimate was −2.00 percentage points relative to BMI < 25 kg/m^2^, with a 95% confidence interval from −5.00 to 1.13. The estimate was therefore imprecise and does not establish an adverse independent effect. Interpretation also depends on the timing and loading conditions of the postoperative echocardiographic assessment.

Postoperative pneumonia occurred in 1.7%, 2.6%, and 10.4% of patients across increasing BMI categories. Although the between-group comparison was not statistically significant, the absolute difference between the BMI ≥ 30 kg/m^2^ and BMI < 25 kg/m^2^ groups was 8.7 percentage points (95% CI, −1.0 to 18.6). This numerically higher rate represents a clinically relevant, hypothesis-generating signal that should be interpreted cautiously because of the small number of events [17]. Mechanical ventilation duration was available in the dataset and did not differ descriptively between patients with and without pneumonia. Atelectasis was not systematically recorded as a separate variable.

Large registry-based analyses have linked increasing BMI to higher operative mortality after cardiac surgery [8]. In the present cohort, in-hospital mortality occurred in one patient, corresponding to an overall estimate of 0.7% with a wide exact 95% confidence interval of 0.0–3.8%. The event count was insufficient for adjusted modeling or reliable between-group inference. The absence of deaths in the higher-BMI groups should not be construed as evidence of a protective effect.

Patient selection is central to the interpretation of these results. Candidacy for robotic mitral valve surgery at our institution is determined through assessment of anatomical and vascular suitability, including prior thoracic surgery, chest wall characteristics, prior thoracic radiotherapy, femoral artery caliber, and aortoiliac disease. Although BMI itself is not regarded as an absolute contraindication, obesity-associated anatomical, vascular, and clinical factors may influence whether an individual patient is offered a robotic approach. The study therefore evaluates outcomes only among patients who were ultimately considered suitable for robotic MVR and does not establish that BMI has no effect on candidacy in the broader population referred for mitral valve surgery.

The obesity findings primarily reflect Class I obesity, as only eight patients had BMI ≥ 35 kg/m^2^ and only one had BMI ≥ 40 kg/m^2^. Preoperative weight optimization may be relevant when elective mitral surgery is considered in patients with severe obesity. Emerging minimally invasive and endovascular weight-management approaches have been described, but their role in preoperative cardiac surgical optimization remains evolving [18].

### Limitations

This study is subject to several limitations. First, its retrospective, single-center design raises the possibility of selection bias and constrains generalizability. Only patients ultimately selected for robotic MVR were included. Because a structured screening log was unavailable, the number and BMI distribution of patients evaluated but not selected for the robotic approach and their reasons for exclusion could not be reliably reconstructed. Accordingly, the findings primarily reflect outcomes among patients considered suitable for robotic MVR and should be extrapolated to the broader population referred for mitral valve surgery with caution. Second, the study covered an eight-year period during which institutional experience, patient selection, and perioperative management may have evolved. Although BMI-group distribution did not differ significantly by calendar year or institutional era, residual temporal confounding remains possible. Third, the sample size was relatively modest, particularly for uncommon postoperative complications, leaving the study underpowered to detect modest differences in low-frequency events such as mortality, stroke, dialysis, and conversion to sternotomy. Fourth, although patients with BMI ≥ 35 kg/m^2^ were represented in the analysis, this subgroup comprised only eight individuals; extrapolation of the present findings to severe obesity should therefore be undertaken with caution. Fifth, baseline characteristics differed across BMI categories, notably age, diabetes, EuroSCORE II, and preoperative LVEF, and the limited number of adverse events precluded robust multivariable adjustment for individual complications. Sixth, the analysis included numerous postoperative endpoints and no prespecified primary endpoint; the study was therefore explicitly treated as exploratory. Although false-discovery-rate adjustment was applied, the possibility of both false-positive and false-negative findings remains. Seventh, the confidence intervals for several adjusted and rare-event estimates were wide, limiting the precision of the estimates. Eighth, 40 of the 48 patients in the BMI ≥ 30 kg/m^2^ group had Class I obesity, whereas only seven had Class II and one had Class III obesity. The findings therefore primarily represent mild obesity and should not be generalized to patients with severe or morbid obesity. The continuous-BMI sensitivity analysis reduces information loss from categorization but does not overcome the sparse representation at the upper end of the BMI distribution. Ninth, although the etiology distribution was available for descriptive reporting, the cohort was predominantly rheumatic (112/146, 76.7%) and should therefore not be generalized to contemporary degenerative mitral regurgitation populations. Tenth, postoperative rhythm surveillance was not based on continuous telemetry throughout the entire hospitalization, and systematic rhythm monitoring was not performed after discharge; therefore, brief or asymptomatic paroxysmal AF episodes may have been underdetected. No formal post hoc power or detectable-effect calculation was performed because of the exploratory retrospective design and multiple outcomes; the confidence intervals are provided to show the precision and range of effects compatible with the data.

## 5. Conclusions

Among patients considered anatomically and clinically suitable for robotic MVR, adjusted analyses did not demonstrate statistically detectable associations between BMI category and the selected operative and early postoperative outcomes. However, the confidence intervals did not establish equivalence and remained compatible with clinically relevant differences for some outcomes. The findings apply only to selected robotic surgical candidates, and the limited representation of severe obesity and low number of major adverse events restrict broader inference. Accordingly, the obesity-related findings primarily apply to Class I obesity and cannot support conclusions regarding severe or morbid obesity. Pneumonia was numerically more frequent in patients with BMI ≥ 30 kg/m^2^, although the estimate was imprecise and hypothesis-generating.

## Figures and Tables

**Table 1:** Preoperative characteristics according to BMI group.

Variable	BMI < 25 (n = 60)	BMI 25–29.9 (n = 38)	BMI ≥ 30 (n = 48)	Overall *p*
Age, years	43.3 ± 16.5	48.0 ± 12.9	53.6 ± 11.5	0.001
Female sex, n (%)	28 (46.7)	18 (47.4)	26 (54.2)	0.757
BSA, m^2^	1.70 ± 0.19	1.84 ± 0.15	1.99 ± 0.14	<0.001
EuroSCORE II, %	0.83 (0.68–1.00)	0.96 (0.72–1.31)	0.97 (0.80–1.64)	0.019
NYHA class				0.091
I	0 (0.0)	1 (2.6)	0 (0.0)	
II	30 (50.0)	11 (28.9)	15 (31.3)	
III	26 (43.3)	19 (50.0)	28 (58.3)	
IV	4 (6.7)	7 (18.4)	5 (10.4)	
Hypertension, n (%)	6 (10.0)	6 (15.8)	10 (20.8)	0.297
Coronary artery disease, n (%)	1 (1.7)	0 (0.0)	1 (2.1)	1.000
Diabetes mellitus, n (%)	5 (8.3)	2 (5.3)	13 (27.1)	0.007
COPD, n (%)	3 (5.0)	2 (5.3)	4 (8.3)	0.829
Chronic kidney disease, n (%)	0 (0.0)	1 (2.6)	1 (2.1)	0.512
Dialysis, n (%)	0 (0.0)	0 (0.0)	1 (2.1)	0.590
Permanent AF, n (%)	6 (10.0)	2 (5.3)	7 (14.6)	0.374
Paroxysmal AF, n (%)	1 (1.7)	1 (2.6)	1 (2.1)	1.000
Preoperative LVEF, %	60 (60–63.5)	60 (55.8–60)	60 (54.8–60)	0.030
LVEDD, mm	51.0 ± 6.2	51.5 ± 7.0	53.0 ± 6.5	0.282
Left atrial diameter, mm	45 (41–52.2)	46 (45–54.8)	47 (43.8–52.2)	0.272
Tricuspid regurgitation				0.107
None	39 (65.0)	29 (76.3)	34 (70.8)	
Mild	18 (30.0)	4 (10.5)	8 (16.7)	
Moderate	3 (5.0)	5 (13.2)	6 (12.5)	
Mitral disease etiology, n (%)				0.380
Rheumatic	44 (73.3)	27 (71.1)	41 (85.4)	
Mixed	12 (20.0)	10 (26.3)	5 (10.4)	
Barlow	2 (3.3)	0 (0.0)	1 (2.1)	
Endocarditis	0 (0.0)	0 (0.0)	1 (2.1)	
Etiology not otherwise specified	2 (3.3)	1 (2.6)	0 (0.0)	

Data are presented as mean ± standard deviation, median (interquartile range), or n (%), as appropriate. Overall comparisons were performed using Welch’s one-way ANOVA for age and BSA, conventional one-way ANOVA for LVEDD, Kruskal–Wallis tests for EuroSCORE II, LVEF, and left atrial diameter, and Fisher’s exact tests for categorical variables. Post hoc comparisons were performed for variables with significant overall differences using Games–Howell tests or Dunn tests with Holm adjustment, as appropriate. Post hoc analysis: age differed between BMI < 25 and BMI ≥ 30 groups (adjusted *p* < 0.001); EuroSCORE II differed between BMI < 25 and BMI ≥ 30 (adjusted *p* = 0.021); preoperative LVEF differed between BMI < 25 and BMI ≥ 30 (adjusted *p* = 0.025). Diabetes mellitus differed between BMI < 25 and BMI ≥ 30 (adjusted *p* = 0.035) and between BMI 25–29.9 and BMI ≥ 30 (adjusted *p* = 0.029). All three BMI groups differed significantly in BSA. Etiology categories were mutually exclusive for descriptive reporting; the overall etiology *p* value was exploratory because of sparse cells. Abbreviations: AF, atrial fibrillation; BMI, body mass index; BSA, body surface area; COPD, chronic obstructive pulmonary disease; LVEF, left ventricular ejection fraction; LVEDD, left ventricular end-diastolic diameter; NYHA, New York Heart Association.

**Table 2:** Intraoperative characteristics according to BMI group.

Variable	BMI < 25 (n = 60)	BMI 25–29.9 (n = 38)	BMI ≥ 30 (n = 48)	Overall *p*
CPB time, min	154.5 (133.2–184.2)	148 (119–181.5)	163 (127.5–202.2)	0.401
Cross-clamp time, min	102 (82.8–123.2)	92.5 (77.2–111.5)	104 (85.5–127.8)	0.234
Prosthesis type, n (%)				0.321
Mechanical	51 (85.0)	33 (86.8)	36 (75.0)	
Bioprosthetic	9 (15.0)	5 (13.2)	12 (25.0)	
Conversion to sternotomy, n (%)	1 (1.7)	1 (2.6)	3 (6.2)	0.525
AF ablation, n (%)	6 (10.0)	3 (7.9)	3 (6.2)	0.926

Data are presented as median (interquartile range) or n (%), as appropriate. Overall comparisons were performed using the Kruskal–Wallis test for continuous variables and Fisher’s exact test for categorical variables. Abbreviations: AF, atrial fibrillation; BMI, body mass index; CPB, cardiopulmonary bypass.

**Table 3:** Postoperative outcomes according to BMI group.

Variable	BMI < 25 (n = 60)	BMI 25–29.9 (n = 38)	BMI ≥ 30 (n = 48)	Unadjusted *p*	FDR-Adjusted q
Ventilation time, h	11 (9.75–12)	10 (8.5–11)	10 (7–12)	0.181	0.786
ICU stay, h	23 (20–25)	23 (20–24)	22.5 (18.75–29.5)	0.635	1.000
Hospital stay, days	7 (6–8)	7 (6–8)	7 (5–9)	0.792	1.000
Drainage, mL	300 (250–350)	250 (250–300)	300 (250–362.5)	0.690	1.000
Red blood cell transfusion, units	1 (0–1)	1 (0–1)	1 (0–1)	0.934	1.000
Postoperative LVEF, %	60 (55–60)	60 (55–60)	55 (53.75–60)	0.038	0.765
New-onset AF, n (%)	7 (11.7)	1 (2.6)	6 (12.5)	0.236	0.786
Temporary pacemaker, n (%)	1 (1.7)	2 (5.3)	1 (2.1)	0.681	1.000
Permanent pacemaker, n (%)	1 (1.7)	1 (2.6)	1 (2.1)	1.000	1.000
Transient cerebrovascular event, n (%)	0 (0.0)	1 (2.6)	1 (2.1)	0.517	1.000
Permanent cerebrovascular event, n (%)	1 (1.7)	0 (0.0)	0 (0.0)	1.000	1.000
Acute kidney injury, n (%)	4 (6.7)	0 (0.0)	4 (8.3)	0.216	0.786
Dialysis, n (%)	1 (1.7)	0 (0.0)	1 (2.1)	1.000	1.000
Pericardial complication, n (%)				0.763	1.000
Pericardial effusion	1 (1.7)	2 (5.3)	1 (2.1)		
Cardiac tamponade	2 (3.3)	0 (0.0)	1 (2.1)		
Pleural effusion, n (%)	3 (5.0)	0 (0.0)	2 (4.2)	0.447	1.000
Infectious complication, n (%)					
Pneumonia	1 (1.7)	1 (2.6)	5 (10.4)	0.108	0.786
Groin infection	0 (0.0)	0 (0.0)	0 (0.0)	—	—
Pneumothorax, n (%)	3 (5.0)	0 (0.0)	0 (0.0)	0.184	0.786
Reoperation for bleeding, n (%)	2 (3.3)	0 (0.0)	2 (4.2)	0.569	1.000
Mortality, n (%)	1 (1.7)	0 (0.0)	0 (0.0)	1.000	1.000
Early readmission, n (%)	0 (0.0)	1 (2.6)	2 (4.2)	0.352	1.000

Data are presented as median (interquartile range) or n (%), as appropriate. Overall comparisons were performed using the Kruskal–Wallis test for continuous variables and Fisher’s exact test for categorical variables. No statistical comparison was performed for groin infection because no events occurred in any BMI group. Pericardial complication was analyzed as a binary composite variable (pericardial effusion or cardiac tamponade vs. neither) in a single 3 × 2 contingency table across the three BMI groups. The Benjamini–Hochberg procedure was applied to the unrounded *p* values across the 20 postoperative endpoints with available statistical comparisons. None met the q < 0.05 threshold; the unadjusted postoperative LVEF *p* value of 0.038 corresponded to q = 0.765. Abbreviations: AF, atrial fibrillation; BMI, body mass index; ICU, intensive care unit; LVEF, left ventricular ejection fraction. For pneumonia, the absolute difference between BMI ≥ 30 kg/m^2^ and BMI < 25 kg/m^2^ was 8.7 percentage points (95% CI, −1.0 to 18.6). Red blood cell transfusion was reported as median units; the proportion receiving at least one unit was 35/60 (58.3%), 20/38 (52.6%), and 27/48 (56.3%) across the BMI groups, respectively. Counts included intraoperative transfusions.

**Table 4:** Effect estimates for clinically selected outcomes and rare events. (**A**) Adjusted median quantile regression; (**B**) rare-event proportions with exact 95% confidence intervals; (**C**) sensitivity analysis with BMI as a continuous variable.

(**A**)				
**Outcome**	**BMI Comparison**	**Adjusted Median Difference**	**Bootstrap 95% CI**	**Bootstrap *p***
CPB time, min	25.0–29.9 vs. <25	0.20	−32.56 to 20.36	0.643
	≥30 vs. <25	7.32	−25.93 to 37.31	0.824
Ventilation duration, h	25.0–29.9 vs. <25	−0.58	−1.85 to 0.00	0.170
	≥30 vs. <25	−0.81	−3.00 to 0.48	0.217
Hospital length of stay, days	25.0–29.9 vs. <25	0.00	−1.33 to 1.00	0.833
	≥30 vs. <25	0.00	−0.76 to 1.26	0.986
Postoperative LVEF, percentage points	25.0–29.9 vs. <25	0.20	−3.60 to 2.79	1.000
	≥30 vs. <25	−2.00	−5.00 to 1.13	0.311
(**B**)				
**Outcome**	**BMI < 25 (n = 60)**	**BMI 25.0–29.9 (n = 38)**	**BMI ≥ 30 (n = 48)**	**Overall (n = 146)**
Conversion to sternotomy	1/60; 1.7 (0.0–8.9)	1/38; 2.6 (0.1–13.8)	3/48; 6.2 (1.3–17.2)	5/146; 3.4 (1.1–7.8)
Transient cerebrovascular event	0/60; 0.0 (0.0–6.0)	1/38; 2.6 (0.1–13.8)	1/48; 2.1 (0.1–11.1)	2/146; 1.4 (0.2–4.9)
Permanent cerebrovascular event	1/60; 1.7 (0.0–8.9)	0/38; 0.0 (0.0–9.3)	0/48; 0.0 (0.0–7.4)	1/146; 0.7 (0.0–3.8)
Dialysis	1/60; 1.7 (0.0–8.9)	0/38; 0.0 (0.0–9.3)	1/48; 2.1 (0.1–11.1)	2/146; 1.4 (0.2–4.9)
In-hospital mortality	1/60; 1.7 (0.0–8.9)	0/38; 0.0 (0.0–9.3)	0/48; 0.0 (0.0–7.4)	1/146; 0.7 (0.0–3.8)
(**C**)				
**Outcome**		**Adjusted Median Change per 5 kg/m^2^ BMI Increase**	**Bootstrap 95% CI**	
CPB time, min		2.91	−10.91 to 14.22	
Ventilation duration, h		−0.50	−1.29 to 0.10	
Hospital length of stay, days		0.00	−0.37 to 0.49	
Postoperative LVEF, percentage points		−0.78	−1.73 to 0.63	

Models included BMI category, age, diabetes mellitus, preoperative LVEF, and procedural year; EuroSCORE II was not included because of overlap with these covariates. BMI < 25 kg/m^2^ was the reference group. Confidence intervals and *p* values were obtained from 2000 bootstrap replications; *p* values used the two-sided bootstrap sign-probability method described in the Methods. All models included 146 patients. Values are n/N; percentage (exact Clopper–Pearson 95% confidence interval). These outcomes were not entered into multivariable models because of the low number of events. Estimates represent adjusted median changes per 5 kg/m^2^ increase in BMI.

## Data Availability

The data supporting the findings of this study are available from the corresponding author upon reasonable request. The data are not publicly available due to privacy and ethical restrictions.

## Supplementary Materials

The following supporting information can be downloaded at https://www.mdpi.com/article/10.3390/jcm15197756/s1. Table S1: Annual distribution of procedures according to BMI category.

## Abbreviations

AF	Atrial fibrillation
ANOVA	Analysis of variance
BMI	Body mass index
BSA	Body surface area
COPD	Chronic obstructive pulmonary disease
CPB	Cardiopulmonary bypass
ICU	Intensive care unit
IQR	Interquartile range
LVEF	Left ventricular ejection fraction
LVEDD	Left ventricular end-diastolic diameter
MVR	Mitral valve replacement
NYHA	New York Heart Association
SD	Standard deviation
TEE	Transesophageal echocardiography

## References

[1] Piché M.-E., Tchernof A., Després J.-P. (2020). Obesity phenotypes, diabetes, and cardiovascular diseases. Circ. Res..

[2] Jensen M.D., Ryan D.H., Apovian C.M., Ard J.D., Comuzzie A.G., Donato K.A., Hu F.B., Hubbard V.S., Jakicic J.M., Kushner R.F. (2014). 2013 AHA/ACC/TOS guideline for the management of overweight and obesity in adults: A report of the American College of Cardiology/American Heart Association Task Force on Practice Guidelines and The Obesity Society. Circulation.

[3] World Health Organization (2000). Obesity: Preventing and Managing the Global Epidemic.

[4] Ghanta R.K., LaPar D.J., Zhang Q., Devarkonda V., Isbell J.M., Yarboro L.T., Kern J.A., Kron I.L., Speir A.M., Fonner C.E. (2017). Obesity increases risk-adjusted morbidity, mortality, and cost following cardiac surgery. J. Am. Heart Assoc..

[5] Donataccio M.P., Vanzo A., Bosello O. (2021). Obesity paradox and heart failure. Eat. Weight Disord..

[6] Elagizi A., Kachur S., Lavie C.J., Carbone S., Pandey A., Ortega F.B., Milani R.V. (2018). An overview and update on obesity and the obesity paradox in cardiovascular diseases. Prog. Cardiovasc. Dis..

[7] Amundson D.E., Djurkovic S., Matwiyoff G.N. (2010). The obesity paradox. Crit. Care Clin..

[8] Mariscalco G., Wozniak M.J., Dawson A.G., Serraino G.F., Porter R., Nath M., Klersy C., Kumar T., Murphy G.J. (2017). Body mass index and mortality among adults undergoing cardiac surgery: A nationwide study with a systematic review and meta-analysis. Circulation.

[9] Burgos L.M., Gil Ramírez A., Seoane L., Espinoza J., Furmento J.F., Costabel J.P., Benzadón M., Navia D. (2021). Is the obesity paradox in cardiac surgery really a myth? Effect of body mass index on early and late clinical outcomes. J. Cardiothorac. Vasc. Anesth..

[10] Kocaoglu A.S., Ovali C. (2025). Early results of female patients who underwent mitral valve surgery through periareolar incision approach. Perfusion.

[11] Russo G., Maisano F., Massaro G., Terlizzese G., Mariano E., Bonanni M., Matteucci A., Bezzeccheri A., Benedetto D., Chiricolo G. (2022). Challenges and Open Issues in Transcatheter Mitral Valve Implantation: Smooth Seas Do Not Make Skillful Sailors. Front. Cardiovasc. Med..

[12] Kitahara H., Patel B., McCrorey M., Nisivaco S., Balkhy H.H. (2017). Morbid obesity does not increase morbidity or mortality in robotic cardiac surgery. Innovations.

[13] Senay S., Cacur O., Bastopcu M., Gullu A.U., Kocyigit M., Alhan C. (2021). Robotic mitral valve operations can be safely performed in obese patients. J. Card. Surg..

[14] Nashef S.A.M., Roques F., Sharples L.D., Nilsson J., Smith C., Goldstone A.R., Lockowandt U. (2012). EuroSCORE II. Eur. J. Cardiothorac. Surg..

[15] Moscarelli M., Bianchi G., Margaryan R., Cerillo A., Farneti P., Murzi M., Solinas M. (2015). Accuracy of EuroSCORE II in patients undergoing minimally invasive mitral valve surgery. Interact. Cardiovasc. Thorac. Surg..

[16] Issa H.M.N., Rahmouni K., Nantsios A., Messika-Zeitoun D., Ruel M., Mesana T., Chan V. (2025). The obesity paradox in patients undergoing surgical repair of degenerative mitral regurgitation. J. Clin. Med..

[17] Xiao P., Song W., Han Z. (2018). Characteristics of pulmonary infection after mitral valve repair in patients with metabolic syndrome and its relationship with blood pressure, blood glucose and blood lipid. Exp. Ther. Med..

[18] Sangiorgi G.M., Cereda A., Porchetta N., Benedetto D., Matteucci A., Bonanni M., Chiricolo G., De Lorenzo A. (2021). Endovascular Bariatric Surgery as Novel Minimally Invasive Technique for Weight Management in the Morbidly Obese: Review of the Literature. Nutrients.

