# Supplementary material for: Isolated Robotic Mitral Valve Replacement Across Body Mass Index Categories: Early Perioperative Outcomes
Source: J Clin Med. 2026 Oct 8;15(19):7756. doi: 10.3390/jcm15197756 (PMC13666070; doi:10.3390/jcm15197756)
Supplement: Supplementary file 1 [file jcm-15-07756-s001.zip › jcm-4542559-supplementary.pdf]

Supplementary Table S1. Annual distribution of procedures according to BMI category

Year	BMI <25 kg/m²	BMI 25.0–29.9 kg/m²	BMI ≥30 kg/m²	Total
2016	8 (33.3)	7 (29.2)	9 (37.5)	24
2017	8 (38.1)	9 (42.9)	4 (19.0)	21
2018	9 (60.0)	1 (6.7)	5 (33.3)	15
2019	7 (50.0)	6 (42.9)	1 (7.1)	14
2020	2 (50.0)	1 (25.0)	1 (25.0)	4
2021	8 (44.4)	4 (22.2)	6 (33.3)	18
2022	9 (42.9)	3 (14.3)	9 (42.9)	21
2023	1 (16.7)	2 (33.3)	3 (50.0)	6
2024	8 (34.8)	5 (21.7)	10 (43.5)	23
Overall	60 (41.1)	38 (26.0)	48 (32.9)	146

Values are n (% within calendar year). Monte Carlo Fisher–Freeman–Halton test across calendar years (1,000,000 resamples): p=0.311.
